# Return to Work After Surgery for Degenerative Cervical Myelopathy: Prospective Data From a Swedish Nationwide Cohort of 789 Patients

**DOI:** 10.1227/neu.0000000000003822

**Published:** 2025-10-17

**Authors:** Victor Gabriel El-Hajj, Marcus Roland Victor Gustafsson, Mateo Tomas Fariña Nuñez, Victor E. Staartjes, Erik Edström, Adrian Elmi-Terander

**Affiliations:** 1 Department of Clinical Neuroscience, Karolinska Institutet, Stockholm, Sweden;; 2 Department of Orthopedic Surgery, Neurosurgery and Spine Surgery, Schulthess Clinic, Zürich, Switzerland;; 3 Machine Intelligence in Clinical Neuroscience & Microsurgical Neuroanatomy (MICN) Laboratory, Department of Neurosurgery, Clinical Neuroscience Center, University Hospital Zurich, University of Zurich, Zurich, Switzerland;; 4 Capio Spine Center Stockholm, Upplands Väsby, Sweden;; 5 Department of Medical Sciences, Örebro University, Örebro, Sweden;; 6 Department of Surgical Sciences, Uppsala University, Uppsala, Sweden

**Keywords:** Return to work, Degenerative cervical myelopathy, Spine surgery, Cervical, Neurosurgery

## Abstract

**BACKGROUND AND OBJECTIVES:**

Degenerative cervical myelopathy (DCM) is a progressive disorder that leads to significant neurological deficits, often requiring surgical decompression to prevent further decline. There are only a handful of studies analyzing return-to-work (RTW) outcomes after cervical spine surgery for DCM. This study seeks to elucidate RTW outcomes and to identify predictors preventing RTW in patients surgically treated for DCM in a nationwide prospective registry.

**METHODS:**

A nationwide cohort analysis was conducted using prospectively gathered data from patients surgically treated for DCM, from the Swedish Spine Registry. Patients with documented postoperative outcomes focusing on RTW rates from 1 to 5 years were included. To identify predictive factors affecting RTW at 1 year postoperatively, separate univariable and multivariable logistic regression models were developed, incorporating demographic, functional and clinical, as well as preoperative and postoperative data and occupational characteristics.

**RESULTS:**

A total of 789 patients were included with an average age of 52 years, with most patients working in moderate intensity jobs and nearly half were on sick leave before surgery. Most surgeries were elective, using an anterior approach. The RTW rate at 1 year was 76%, separating into 54% who had resumed full-time employment and 23% who had returned to a part-time capacity. In this cohort, 24% had not returned to work at the 1-year mark. Older age, physically demanding work, higher preoperative Neck Disability Index Score, reduced walking distance, and sickness benefits were significant predictors of a lack of RTW.

**CONCLUSION:**

75% of the patients surgically treated for DCM returned to work within 1 year. Higher age, physically demanding work, higher Neck Disability Index Score, and full-time sickness benefits were all associated with a decreased likelihood of RTW. Recognizing these risk factors can help identify patients who may benefit from additional physical therapy, behavioral interventions, counseling, or work-place adjustments to support RTW.

## Graphical abstract

**Figure:**
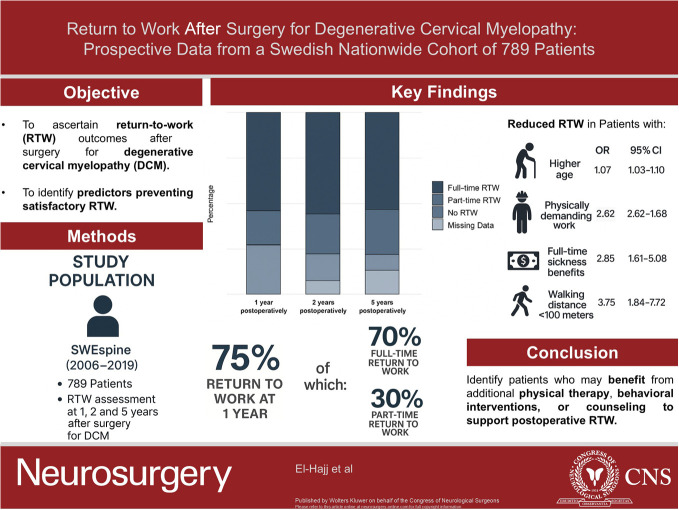

**ABBREVIATIONS:**
DCM: degenerative cervical myelopathy
NDI: Neck Disability Index
RTW: return to work
Swespine: Swedish Spine Registry.

Degenerative cervical myelopathy (DCM) is a progressive disorder that leads to significant neurological deficits, including sensorimotor decline, profoundly impairing quality of life.^1^ The clinical presentation of DCM comprises a wide range of symptoms including neck pain and radicular pain, numbness of the upper extremities, impairments of fine motor skills developing to cases with severe upper and lower limb paresis, progressive gait instability, and bladder dysfunction.^2,3^ Delayed diagnosis and late referral due to poor awareness and incomplete neurological evaluation can delay the assessment for surgical decompression, leading to poorer outcomes and risk of developing permanent life-long neurological disability.^4-6^

Nowadays, DCM stands as the most prevalent cause of spinal cord impairment among adults aged older than 55 years.^7-9^ Beyond the considerable personal suffering it inflicts, DCM also imposes a substantial economic burden on society, largely attributable to lost productivity. Studies on the impact of chronic neuropathic pain identify a considerable socioeconomic cost secondary to loss of ability to work.^10^ In many cases, surgical intervention becomes necessary to alleviate symptoms and restore neurological function. Accordingly, return to work (RTW) emerges as a relevant measure of surgical success. Although surgery for DCM frequently results in alleviation of neurological deficits and pain associated with cervical myelopathy, it is noteworthy that not all patients are able to RTW postoperatively.^11-13^

Few studies have assessed RTW following surgery for DCM,^8,14^ and the predictors of RTW remain incompletely understood. Using a nation-wide prospective registry, the aim of this study was to elucidate predictors preventing RTW in a cohort of patients surgically treated for DCM.

## METHODS

### Study Design

This analysis used data from the Swedish Spine Registry (Swespine), a prospective multicenter registry established to evaluate surgical outcomes for degenerative spinal conditions in Sweden.^15-21^ Swespine has a coverage of 98% (46 of 47 spinal clinics), the completeness is approximately 75% (30%-90%), and the 1-year follow up is performed in approximately 75%, covering all types of spinal surgical procedures. Swespine uses standardized protocols for enrollment and data collection. Data were extracted from patients who had been surgically treated for DCM and where postoperative outcomes were available. These included patient-reported outcome measures, such as the Numeric Rating Scale for neck and arm pain severity and the Neck Disability Index (NDI). This study was approved by the Swedish National Ethics Review Authority (Dnr 2020-00193, and 2021-04773), which waived the requirement for informed consent in accordance with Swedish legislation.

### Patient Population and Variables

Adult patients of working age who underwent surgery for DCM between 2006 and 2019 were eligible for inclusion. Although Sweden does not have a fixed retirement age, 65 years was still seen as the standard retirement age in Sweden during the study period. The pension is largely based on life-time earnings, and, consequently, there is an incentive to work beyond the reference age for retirement. To qualify for Swedish sickness-benefit, the individual must be insured in the Swedish social security system, have worked full time for at least 6 months, have a doctor assess and find a reduced ability to work, and finally, the Swedish Social Insurance Agency must approve the benefit. The compensation is 80% of the preceding income up to about 60 000 USD per year and declines after 12 months. Patients aged older than 64 years were hence excluded from the analysis. Patients who were unemployed at the time of surgery were excluded from the analysis. Patients with incomplete records, precluding analysis of RTW and its predictors, were excluded. Work intensity was defined as low (equivalent to office duties), moderate (a combination of administrative and physical duties, for example, working in a store), and high (heavy physical labor in industry, agriculture of transport sections). Myelopathy was assessed using the European myelopathy score.

### Outcomes

The primary outcome of this study was to assess the rate of RTW within the first year after surgery and to identify which factors independently predict failure to RTW. Secondary outcomes included evaluating RTW rates at 2 and 5 years postoperatively, measuring the time until RTW was achieved. RTW was classified as complete, partial, or no RTW, according to whether the predisease work status was achieved again or not.

### Statistical Analysis

Continuous variables were described using means and SDs, whereas categorical variables were presented as frequencies and percentages. To identify factors associated with RTW at 12 months after surgery, separate univariable logistic regression models were constructed. These models accounted for a comprehensive range of preoperative, intraoperative, and occupational factors. To identify independent predictors, a multivariable logistic regression model for 12-month RTW was then established based on univariable significance (univariable *P* ≤ .10) and excluding multicollinear variables. Statistical significance was determined with a *P* of ≤ .05. Statistical analyses were performed using R statistical software.

## RESULTS

### Preoperative Clinical Characteristics and Surgical Procedures

**FIGURE 1.**
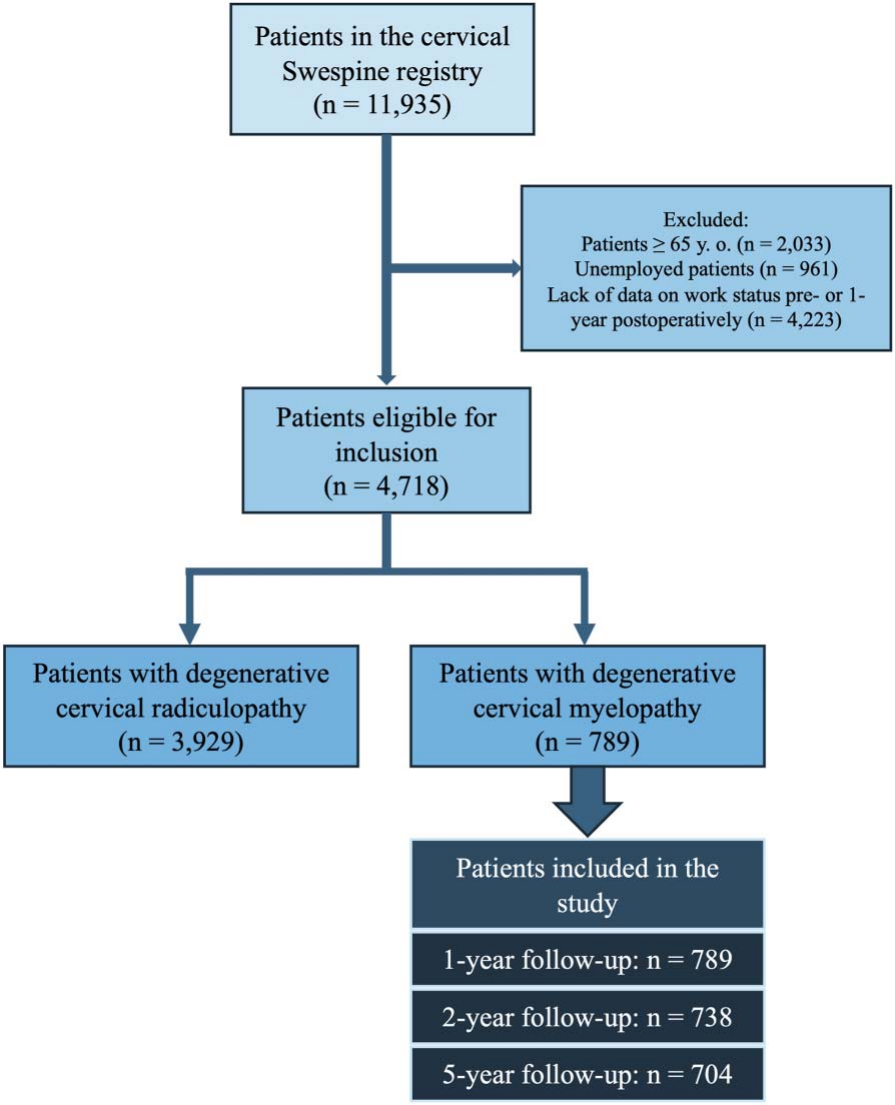
Flowchart.

Among the 789 individuals included in the study (Figure 1), the average age was 52 years, men comprised 52% of the cohort, and the mean body mass index was 27.2. Regarding occupational demands as classified in the Swespine registry, the largest proportion of patients (41%) were engaged in jobs of moderate intensity, whereas 32% held positions classified as low intensity and 26% as high intensity. A history of previous cervical spine surgery was reported by 8.7% of participants. Regarding work status before surgery, half of the patients were employed full time, whereas 47% were on sick leave because of symptoms affecting the neck or arm. At the time of surgery, 19% of the cohort were receiving full-time sickness benefits and 12% were on part-time sickness benefits. The duration of neck pain varied, with 15% experiencing symptoms for more than 2 years, 23% for 1 to 2 years, and 17% for less than 3 months. For those reporting arm pain, 19% had symptoms lasting over 2 years, 31% for 1 to 2 years, and 13% for less than 3 months. Among patients using pain medication, 39% reported daily use, 31% occasional use, and 8.6% used opioids. Functional limitations were common, with 29% experiencing difficulty walking distances more than 500 m and 78% reporting impairments of fine motor skills. Most procedures were elective (95%), with the anterior approach used in 71% of the cases. Diskectomy was the most frequently performed surgery (56%), followed by laminectomy (29%), and fixation was performed in 81% of surgeries (Table 1).

**TABLE 1.** Preoperative Clinical Characteristics and Surgical Procedures

Variable	N = 789
Age	52.25 (7.73)
Body mass index	27.20 (4.28)
Male sex	411 (52%)
Smoker	121 (15%)
Previous cervical spine surgery	69 (8.7%)
Work intensity	
Low	255 (32%)
Moderate	325 (41%)
High	209 (26%)
Preoperative sick leave	
None	394 (50%)
Due to neck/arm issues (full-time)	278 (35%)
Due to neck/arm issues (part-time)	97 (12%)
Due to other cause	20 (2.5%)
Sickness benefit	
None	550 (70%)
Full-time sickness benefit	147 (19%)
Part-time sickness benefit	92 (12%)
Preoperative neck pain duration	
<3 mo	131 (17%)
3-12 mo	44 (5.7%)
1-2 y	175 (23%)
>2 y	120 (15%)
No neck pain	305 (39%)
Preoperative arm pain duration	
<3 mo	98 (13%)
3-12 mo	47 (6.1%)
1-2 y	239 (31%)
>2 y	151 (19%)
No arm pain	241 (31%)
Preoperative pain medication use	
None	237 (30%)
Daily	307 (39%)
Occasional	245 (31%)
Preoperative opioid use	68 (8.6%)
Walking distance	
<100 m	112 (15%)
100-500 m	104 (14%)
0.5-1 km	119 (15%)
>1 km	433 (56%)
Preoperative impairments of fine motor skills	599 (78%)
Type of procedure	
Elective	691 (95%)
Nonelective	37 (5.1%)
Approach	
Anterior	561 (71%)
Posterior	228 (29%)
Procedure	
Diskectomy	443 (56%)
Laminectomy	228 (29%)
Corpectomy	104 (13%)
Arthroplasty	14 (1.8%)
Use of fixation	639 (81%)
Highest level operated	
C2	4 (0.5%)
C3	12 (1.6%)
C4	147 (20%)
C5	211 (29%)
C6	304 (41%)
C7	62 (8.0%)
Number of operated levels	1.92 (0.97)

### Primary Outcome—RTW

**FIGURE 2.**
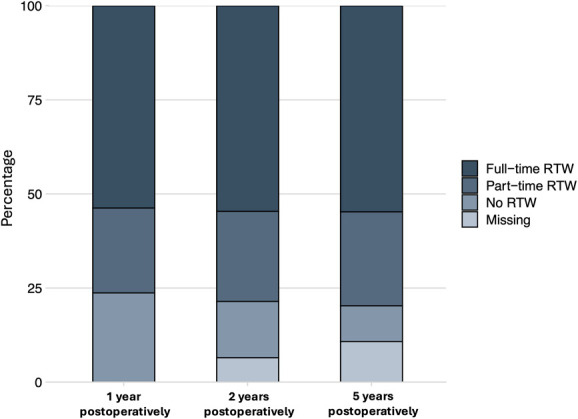
RTW analysis with 1, 2, and 5 years of postoperative follow-ups. RTW, return to work.

Of the 789 patients included in the study, 45% were able to RTW within 3 months after their surgery for DCM, whereas an additional 29% returned between 3 and 6 months postoperatively. At the 1-year mark, 54% of patients were working full-time, 23% worked part-time, and 24% did not work. Overall, the RTW rate at 1 year was 76%, with 70% of those working full-time and 30% part-time. Notably, 22% of patients who returned to work did so with work adjustments. Overall, most individuals (98%) who returned to work did so within the first year after surgery. Two years after surgery, 55% of patients worked full time, 24% part time, and 15% had not returned to work. At the 5-year follow-up, 55% worked full time, 25% part-time, and 9.5% did not work (Table 2 and Figure 2).

**TABLE 2.** RTW Analysis With 1, 2 and 5 Years of Postoperative Follow-up

Variable	N = 789
Postoperative sick leave	
<3 mo	349 (45%)
3-6 mo	227 (29%)
6-9 mo	60 (7.7%)
9-12 mo	47 (6.0%)
≥1 y	96 (12%)
Work status at 1-y postoperatively	
Full-time RTW	424 (54%)
Part-time RTW	178 (23%)
No RTW	187 (24%)
Work status 2 y postoperatively	
Full-time RTW	431 (55%)
Part-time RTW	189 (24%)
No RTW	118 (15%)
Missing	51 (6.5%)
Work status 5 y postoperatively	
Full-time RTW	432 (55%)
Part-time RTW	197 (25%)
No RTW	75 (9.5%)
Missing	85 (11%)

RTW, return to work.

### Factors Predicting Failure to RTW

Older age was independently associated with lower RTW (odds ratio [OR] = 1.07, 95% CI = 1.03 to 1.10, *P* < .001). Job demands also played a crucial role: The multivariable analysis reveals that patients employed in high-intensity occupations had 2.6-fold greater odds of not returning to work (OR = 2.62, 95% CI 1.40 to 4.98, *P* < .001); those in moderately demanding jobs also had an elevated risk of not returning to work (OR = 1.73, 0.96 to 3.15, *P* < .001), compared with individuals in less physically demanding roles, however, only in the univariable analysis. Sickness benefits were another important factor associated with lower RTW: Patients receiving full-time sickness benefits were more likely to not RTW postoperatively (OR = 1.27, CI 1.61 to 5.08, *P* < .001). The univariable analysis identified arm pain for 1 to 2 years but not for shorter or longer as a predictor of failing to RTW. However, this finding was not significant in the multivariable analysis. Similarly, the length of neck pain before surgery did not show a significant association. Patients with a preoperative walking distance of less than 500 m had poorer RTW rates (OR = 2.82, CI 0.77 to 11.6, *P* < .033). Finally, higher preoperative NDI scores, indicating greater disability, were significantly associated with a lower probability of RTW (OR = 1.03, CI 1.01 to 1.05; *P* < .0138; Table 3).

**TABLE 3.** Predictors of No RTW

Characteristic	Univariable *P*-value	OR	95% CI	Multivariable *P*-value
Age	**<.001**	1.07	1.03, 1.10	**<.001**
Body mass index	**.007**	1.01	0.97, 1.07	.56
Male sex	.69			
Smoker	**.002**	1.29	0.72, 2.31	.39
Previous cervical spine surgery	.43			
Work intensity (physically demanding)				
Low	REF	REF	REF	REF
Moderate	**.001**	1.73	0.96, 3.15	.070
High	**<.001**	2.62	1.40, 4.98	**.003**
Preoperative sick leave				
None	REF	REF	REF	REF
Due to neck/arm issues (full-time)	**<.001**	1.27	0.70, 2.29	.42
Due to neck/arm issues (part-time)	.080	0.98	0.44, 2.12	.96
Due to other cause	.087	0.91	0.23, 3.22	.88
Sickness benefits				
None	REF	REF	REF	REF
Full-time sickness benefits	**<.001**	2.85	1.61, 5.08	**<.001**
Part-time sickness benefits	**<.001**	1.97	0.94, 4.13	.072
Preoperative neck pain duration				
<3 mo	REF	REF	REF	REF
3-12 mo	.38	0.97	0.27, 3.66	.96
1-2 y	.070	1.53	0.42, 6.02	.53
>2 y	.055	1.56	0.44, 5.92	.50
No neck pain	.31	1.34	0.36, 5.36	.67
Preoperative arm pain duration				
<3 mo	REF	REF	REF	REF
3-12 mo	.38	1.33	0.39, 5.13	.66
1-2 y	**.033**	2.82	0.77, 11.6	.13
>2 y	.11	1.59	0.43, 6.61	.50
No arm pain	.60	2.10	0.52, 9.30	.31
Preoperative pain medication use				
None	REF	—	—	—
Regularly	.067	—	—	—
Occasionally	.49	—	—	—
Preoperative opioid medication use	.97	—	—	—
Walking distance				
>1 km	REF	REF	REF	REF
0.5-1 km	**.008**	1.43	0.72, 2.77	.29
100-500 m	**<.001**	3.30	1.68, 6.50	**<.001**
<100 m	**<.001**	3.75	1.84, 7.72	**<.001**
Preoperative impairments of fine motor skills	**.001**	0.69	0.36, 1.34	.26
Type of procedure				
Elective	REF	—	—	—
Nonelective	.78	—	—	—
Surgical approach				
Anterior	REF	—	—	—
Posterior	**.010**	0.78	0.35, 1.70	.53
Surgical procedure				
Diskectomy	REF	REF	REF	REF
Laminectomy	**.010**	0.78	0.35, 1.70	.53
Corpectomy	.37	1.46	0.73, 2.86	.28
Arthroplasty	.98	—	—	—
Fixation surgery	**.027**	0.47	0.21, 1.03	.060
Highest operated level^a^	**.025**	0.92	0.69, 1.22	.54
Number of operated levels	**.005**	1.00	0.76, 1.32	>.99
Preoperative NRS neck pain score	**.028**	0.92	0.82, 1.04	.18
Preoperative NRS arm pain score	**.019**	1.00	0.90, 1.11	.97
Preoperative EQ-5D index	**<.001**	0.89	0.37, 2.12	.78
Preoperative EQ-VAS score	**<.001**	—	—	—
Preoperative NDI	**<.001**	1.03	1.01, 1.05	**.013**
Preoperative European myelopathy score	**<.001**	0.92	0.81, 1.04	.20

EQ-5D, EuroQOL five dimensions questionnaire; EQ-VAS, EuroQOL visual analogue scale; NDI, Neck Disability Index; NRS, Numeric Rating Scale; OR, odds ratio; REF, reference level; RTW, return to work. Bold entries signify a *P* value of < .05.

a The highest operated vertebral level was encoded as a continuous variable by assigning each cervical vertebra (C2 to C7) a numerical value from 1 to 6, respectively.

## DISCUSSION

This study evaluated the predictors and outcomes associated with RTW in a nationwide Swedish cohort of 789 patients surgically treated for DCM. To our knowledge, this is one of the largest multicenter series focusing on RTW rates after surgery for DCM. The results show that 76% of patients were able to resume work within 1 year postoperatively, with this figure stabilizing at 80% at the 5-year follow-up. Almost all, 98%, who returned to employment did so within the first year. These findings are consistent with previous studies, which have shown that most patients who RTW after cervical spine surgery for DCM do so within the first year, with the strongest predictors of RTW being fewer preoperative sick days and improvements in functional status.^22,23^ Similar to our findings, another Scandinavian study performed on a nationwide Norwegian registry, performed by Lønne et al,^8^ reported RTW rates of 65% at 12 months and 75% at 36 months after surgery for DCM. Overall, available data on elective surgery in the cervical spine show greater RTW, suggesting that the subgroup of patients with DCM face greater challenges in returning to work.^24^

In this study, several preoperative factors were found to significantly affect RTW outcomes. Higher age, more physically demanding work, higher preoperative NDI score, reduced walking distance, and full-time sickness benefits were all associated with a decreased likelihood of returning to work. These findings are consistent with recent meta-analyses and cohort studies, which have also repeatedly identified age, work intensity, preoperative work status, and baseline disability as key determinants of RTW after cervical spine surgery.^13,22,23,25,26^ To address some of the factors, higher age was revealed as one of the most important factors predicting failure of RTW and is consistent with previous studies.^11,14,27,28^ On the other hand, young age is a predictive factor for favorable RTW outcomes.^24^ Our study also showed that the duration of preoperative arm pain was a predictive factor for not returning to work after cervical spine surgery for DCM. Pain after the surgery is often related with lower RTW rates, as reported by Bergin et al.^22^ The reported impact of pain after surgery on RTW was also supported in this study by the association between greater NDI scores and poorer RTW rates. In addition, our finding of a negative effect of reduced walking distance on RTW resonates with the results from other studies that have found that preoperative neurological and functional impairment predicts postoperative outcome in patients undergoing surgery for DCM.^29^

Interestingly, after adjusting for other factors in the multivariable analysis, surgical approach (anterior vs posterior) did not act as a significant predictor of RTW. Although these findings oppose the ones published by Chanbour et al,^14^ they are in line with a randomized clinical trial conducted by Ghogawala et al,^30^ that showed no significant differences in RTW between anterior and posterior cervical surgeries for cervical spondylosis.

The data further demonstrated that patients receiving full-time sickness benefits were substantially less likely to RTW, with an odds ratio of 2.85 (*P* < .001). This finding is consistent with the results reported by Huysmans et al^31^ and Khan et al,^32^ who observed similar patterns in both European and American social welfare contexts in lumbar spine surgery patients. Although social welfare programs provide essential support, they may inadvertently discourage RTW when prolonged benefits are readily available. Physically demanding jobs were also associated with a markedly increased risk of not returning to work (OR: 2.62, *P* < .001), echoing the findings of Singh et al,^33^ who reported lower RTW rates among individuals in physically demanding jobs. Similarly, Lønne et al^8^ found that having less than 90 days of sick leave before surgery and a higher level of education were strong predictors of successful RTW. Studying elective cervical spine surgery in the United States, Chanbour et al^14^ also identified more physically demanding work as a major determinant of RTW, consistently linked to lower rates of return. These results suggest that socioeconomic and systemic factors can act as obstacles to RTW, independent of clinical recovery.

### Limitations

This study is based on prospectively collected registry data from the Swedish spine registry. Consequently, the study is limited to the data contained therein. Even though the national coverage and response rates are good (98% and 75%, respectively) and the data therefore may be taken to accurately represent the Swedish population, it may not hold true for other populations. Especially since the RTW analysis performed in this study implicates the Swedish Social Insurance Agency, which in its function may differ drastically from similar agencies in other countries. Thus, the external validity of these results in other populations and retirement systems need further investigation. Finally, variables that are not accounted by the Swespine registry may have acted as a source of confounding bias, interfering with the results of the analysis.

## CONCLUSION

Three-quarters of the patients surgically treated for DCM returned to work within 1 year. Higher age, physically demanding work, higher NDI Score, and full-time sickness benefits were independently associated with a decreased likelihood of RTW. Recognizing these risk factors can help identify patients who may benefit from additional physical therapy, behavioral interventions, counseling, or work-place adjustments to support RTW.
